# TOWARD DEVELOPING PATIENT-ORIENTED WEBSITES FOR EARLY DETECTION AND MANAGEMENT OF FRAILTY

**DOI:** 10.1093/geroni/igae098.2774

**Published:** 2024-12-31

**Authors:** Seohyeon Chung, Xiaowei Song

**Affiliations:** Fraser Health Authority, Edmonton, Alberta, Canada; Fraser Health Authority, Surrey, British Columbia, Canada

## Abstract

Understanding frailty status of older adults before a doctor’s visit can result in more effective care planning, where web technology can play role. We conducted a study engaging community-dwelling older adults and caregivers to better understand their perspectives about developing patient-oriented frailty websites. Our objectives are to describe the project activities and share the insights learned from potential end-users of the website. We first organized an online educational symposium consisting of six invited talks on patient-oriented frailty management given by experts of frailty and older adult care. Audience of the symposium was recruited through newsletters and academic, community, and patient association platforms. Clinicians, researchers, caregivers, and community-dwelling older adults across Canada participated (n=300). Members from the symposium (n=14) participated in two virtual discussion sessions with open-ended questions. Discussions highlighted aspects, including the function, usage, and feasibility of a frailty website; security and privacy of online health data; patient-physician interactions; and existing technologies and resources supporting patient-oriented frailty management for successful aging in place. Our findings revealed the needs, barriers, and possible solutions with developing patient-oriented frailty websites. The effort towards building frailty websites that patients can use will benefit future development and implementation to identify people at risk of frailty and facilitate real-time monitoring for personalized and preventative care.

